# Lichens as bio-monitors of polycyclic aromatic hydrocarbons: Measuring the impact of features and traffic patterns

**DOI:** 10.1016/j.heliyon.2023.e20087

**Published:** 2023-09-13

**Authors:** Diana Marcela Uribe, Lina María Ortega, Marco Tadeu Grassi, Rafael Garrett Dolatto, Nazly Efredis Sánchez

**Affiliations:** a Programa de Ingeniería Ambiental, Universidad del Cauca, carrera 2 #15N, Popayán, Cauca, Colombia; b Department of Chemistry, Universidade Federal do Paraná, Jardim das Américas, Caixa Postal 19032, CEP 81531-980, Curitiba, Brazil; c Departamento de Ingeniería Ambiental y Sanitaria, Universidad del Cauca, Carrera 2 #15N, Popayán, Cauca, Colombia

**Keywords:** Air pollution, Speed control devices, Road gradient, Carcinogenic equivalence sum

## Abstract

The role of road characteristics, including gradient and speed control devices, in influencing emission dynamics remains to be fully elucidated. Most studies have focused on fuel consumption as an indirect indicator of sector emissions instead of directly quantifying specific pollutants, like polycyclic aromatic hydrocarbons (PAHs). This research approach is often due to the complexities involved in capturing these pollutants and their subsequent analysis. Bio-monitors, such as lichens, offer an economically viable method. Their wide distribution across various habitats enables the comparison of PAH levels in diverse environments. Against this background, The present work analyses the ability of tropical lichens to indicate the effect that traffic patterns and geometric design features of roads (traffic activity, road gradient, traffic control devices, and vehicular speed) have on the emission of PAH concentration. Results showed that PAHs in lichens strongly correlated with the road gradient (Spearman correlation, p<0.005 with R=0.98). Each 1% increase in road gradient implies a rise of 24 ng_PAH_/g_Lichen_ in National Road. Additionally, a trend coherent of PAH concentration with the vehicle speed profile was observed on Panamericana Road. Speed control devices were associated with higher concentrations of PAHs due to acceleration and braking actions that increment fuel consumption. Finally, the results evidenced that lichens helped determine the source of aromatics and their carcinogenic potential using the diagnostic ratio of PAHs and the carcinogenic equivalence sum, respectively.

## 1 Introduction

Polycyclic aromatic hydrocarbons (PAHs) are considered ubiquitous compounds able to be transported through long distances and which can be deposited in the most remote environments on Earth [1,2]. Furthermore, human exposure to PAHs is considered a health concern since numerous of these compounds are classified as carcinogenic [3]. Consequently, monitoring PAHs in different environments is becoming a public health requirement worldwide. The United States Environmental Protection Agency (US-EPA) determined 16 of them as priority pollutants for environmental monitoring: Naphthalene (Nap), acenaphthylene (Acy), acenaphthene (Ace), fluorene (Flu), anthracene (Ant), phenanthrene (Phe), chrysene (Chry), pyrene (Pyr), benzo(*a*)pyrene (BaP), fluoranthene (Fluo), benzo(*b*)fluoranthene (BbF), benzo(k)fluoranthene (BkF), benzo(*a*)anthracene (BaA), dibenzo(a,h)anthracene (DahA), and benzo(g,h,i)perylene (BghiP), indeno [1,2,3-cd] pyrene (IP).

A source of PAH emissions in urban areas is vehicle traffic, which may present public health risk [4]. This, consecutively, depends on road characteristics, e.g., gradient and speed control devices. Recently, a few studies have quantified the influence of different vehicular traffic patterns and the characteristics of the roads on atmospheric pollutant emissions [5,6]. The geometric design commonly provides elements like intersections, cross-section elements, horizontal curvature, among others, of the roadway; this design primarily focuses on providing safety and maximum traffic efficiency at a reasonable cost. Nonetheless, environmental and health considerations are secondary, if not neglected, constraints when a road and its elements are designed.

Although stakeholders define strategies to reduce air pollution from transportation systems, e.g., eco-driving courses and intelligent transportation systems, very little attention has been drawn to the proper geometric design and; thus, traffic patterns based on environmental factors, even though this can have a considerable effect on air pollution [7].

Research on the effect of traffic patterns on emissions has mainly focused on simulation and experimental data on pollutant dispersion [8]. Moreover, models are used primarily to establish the effect of different factors on fuel consumption to predict pollutant emissions [7,[9], [10], [11]]. Some studies are devoted to the analysis of geometric designs related to emissions of specific pollutants; most of them address greenhouse gases (e.g., CO_2_) and/or criteria air pollutants, e.g., CO, particulate matter (PM), NO_2,_ and SO_2_ rather than on carcinogenic contaminant [12]. To the authors’ knowledge, no bibliographic information is aimed at explicitly assessing the impact of road characteristics and their influence on PAH emissions.

In this context, further studies are required on specific pollutants from traffic emissions and the effect of geometric characteristics of roads to complement the strategies followed to minimize air pollution in urban and rural areas with traffic activity. Additionally, it is necessary to consider the pollutants related to a high impact on public health.

The need for more information on the impact of road design variables on the environment and human health could result from the requirement of high-cost equipment for its assessment. This may represent a limiting factor for pondering toxic pollutants as an essential criterion in the evaluation and consequences of future interventions on road infrastructure, principally in developing or low-income countries.

Lichens have been used as bio-monitors for PAHs in the atmosphere based on their ability to absorb pollutants directly from the air [[13], [14], [15]]. Furthermore, several studies have shown that lichens are efficient accumulators of PAHs distributed in gas and solid phases [[16], [17], [18]]. Specifically, communities of lichens of the family Parmeliaceae have been successfully used as bio-indicators of inorganic and organic pollutants and for evaluating the environmental impact of emissions sources, e.g., road traffic [[19], [20], [21]]. Notwithstanding, to our knowledge, no work has previously been published analysing the effectiveness of lichens for PAH level bio-monitoring in relation to geometric design features, speed control devices, and specific traffic patterns, such as vehicle density. On account of that, the present work aims to evaluate the influence of these variables on the PAH concentrations measured in different species of tropical lichens collected in two sectors: Pan-American Highway (PR) and National Highway No. 20 (NR), in Popayan, Colombia. In addition, the concentration measured in lichens was used to study individual traffic-related PAHs and their toxicity through carcinogenic equivalence sum (KE) based on the toxic equivalence method (commonly called TEQ) and their specific source by isomer ratios.

The results presented here show the usefulness of lichens in determining the specific concentrations of pollutants and their sources. This could lead to obtaining experimental data to strengthen modelling studies and evaluate the geometric designs of roads, to define the best strategies for a healthier environment.

## 2 Materials and methods

### 2.1 Study area

Thirty samples of lichens in trees were collected in April 2019 in two sites. Each site was defined as a section of the road, about 900 m long, in the municipality of Popayan. These small transects were determined with a similar growing environment of lichens to avoid interferences in the interpretation of results. The impact of other anthropogenic sources on air pollution was negligible at both sites, so the measured PAHs could be attributed to vehicle emissions. Selected trees for sampling belong to the family Bignoniaceae, as can be observed in the inventory of trees in the city [22].

Popayán (1760 m.a.s.l.), with a population of 260,000 people, is the capital of the Department of Cauca, located in the southwest of Colombia in the Andean Region, between the Western and Central Mountain Range. This area has deficient industrial activity, so its primary pollutant source corresponds to exhaust emissions and, to a lesser extent, open burning or indoor and outdoor domestic activities such as biomass combustion for cooking.

**Fig. 1:**
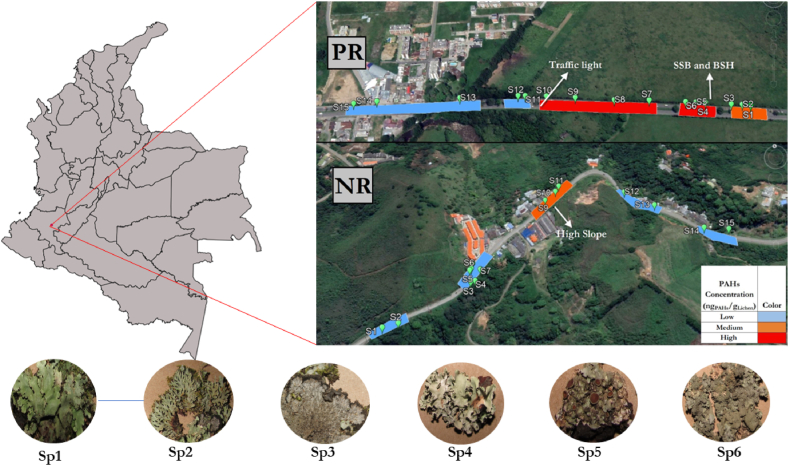
Location of sampling sites (NR and PR) with qualitative levels of PAH associated with lichens and photographs of lichen species collected. Sp1: *Hypotrachyna rhabdiformis (Kurok.) Hale*; Sp2: *Heterodermia obscurata (Nyl.) Trevis*; Sp3: *Parmeliella* sp.; Sp4: *Parmotrema perlatum*; Sp5: *Hypotrachyna* sp.; Sp6: *Punctelia colombiana Sérus*.

The lichen samples were collected from two sites. The first site was located along the Panamericana Road (PR), which is part of the Pan-American Highway, an important network of roads stretching across different American countries. The second site corresponds to the national route No. 20 (NR), which connects the Departments of Cauca and Huila (Fig. 1).

A background site would help better identify the cause and effect of traffic emissions on lichen-associated PAH levels, but it was not considered in the present paper. It is, therefore, recommended for future works.

### 2.2 Road feature and traffic data

To analyze the impact of motor vehicles on PAH levels measured from lichen samples, the volume of vehicle traffic on each road was measured continuously for seven days, 24 per day, in April 2019. To achieve this aim, HD 720mp cameras, brand DAHUA were used, whose recordings were later analyzed to conduct the vehicle count.

In this work, an adaptation of classification rules for motor vehicles of the Federal Highway Administration Research and Technology of the United States Department of Transport (FHWA) has been carried out to characterize the type of vehicles traveling on the selected roads [23]. This classification has been used in previous scientific works [24] and adopted by the Ministry of Transport in Colombia. It includes motorcycles, automobiles, buses, small trucks (C2P-two axle), big trucks (C2G-two axle), heavy vehicles such as Three-axle Truck (C3), Four-axle Truck (C4), Five-axle Truck (C5) and Six or More axle Truck (>C5).

Google Earth Pro was used to determine the elevation profile and gradients of studied zones in NR, as used by Ref. [25]. Transects were split into small segments according to similitude in their patterns for vehicle speed in PR and gradient in NR. PAH concentrations at nearby sampling sites were clustered.

Three vehicles (two public service passenger cars and one sport utility vehicle) were used to obtain the driving speed of three different drivers in PR. A dash camera Vantrue N1pro Full High Definition with a GPS receiver module equipped with a memory card was used to gather the studied trajectories. Geographical Position System (GPS) can check vehicle location/speed/route on Map through Vantrue Player Software. GPS – camera data was recorded on the memory card and post-processed with MATLAB software. All speed profiles of trips (n: 20) were included in this study with red and green traffic lights. Car drivers were not informed of the present study or received instruction not to cause a disturbance in their typical driving behavior. The track in the PR segment was chosen with traffic lights and calming traffic measures (small speed bumps-SSB, big speed humps-BSH) (Table 1). Concerning NR, data was determined for the corridor with a positive gradient, which would cause a higher impact on air pollution. Similar methods for determining vehicular flow dynamics were implemented by Ref. [26].

**Table 1:** Characteristics of the roads and speed control devices.

Road	General characteristics	Number of speed hump or speed bump^1^	Number of traffic lights
NR	Two-way vehicular road with two-lane Track with a bend Gradient interval: 2.6 to 12.8	0	0
PR	Two-way vehicular road with four-lane Linear track Gradient <3% on average	7 pairs of SSB^2^, 2 BSH^2^	1

^1^Control traffic devices were found only in one direction of the PR transect.

^2^SSB: small speed bumps, BSH: Big speed humps.

Table 1 shows the road characteristics of PR and NR. The main difference between both transects is that PR has speed control devices, a linear track, and a larger number of lanes, whereas NR has a higher gradient.

#### 2.2.1 Traffic in the study area

Light-duty vehicles in Colombia primarily use gasoline as fuel, while buses ranging from C2 to greater than C5 use diesel as their primary fuel source. According to the Transport Ministry in Colombia [27], around 75% and 24% of vehicles for passenger transportation across municipalities use diesel and gasoline as fuel, respectively.

**Fig. 2:**
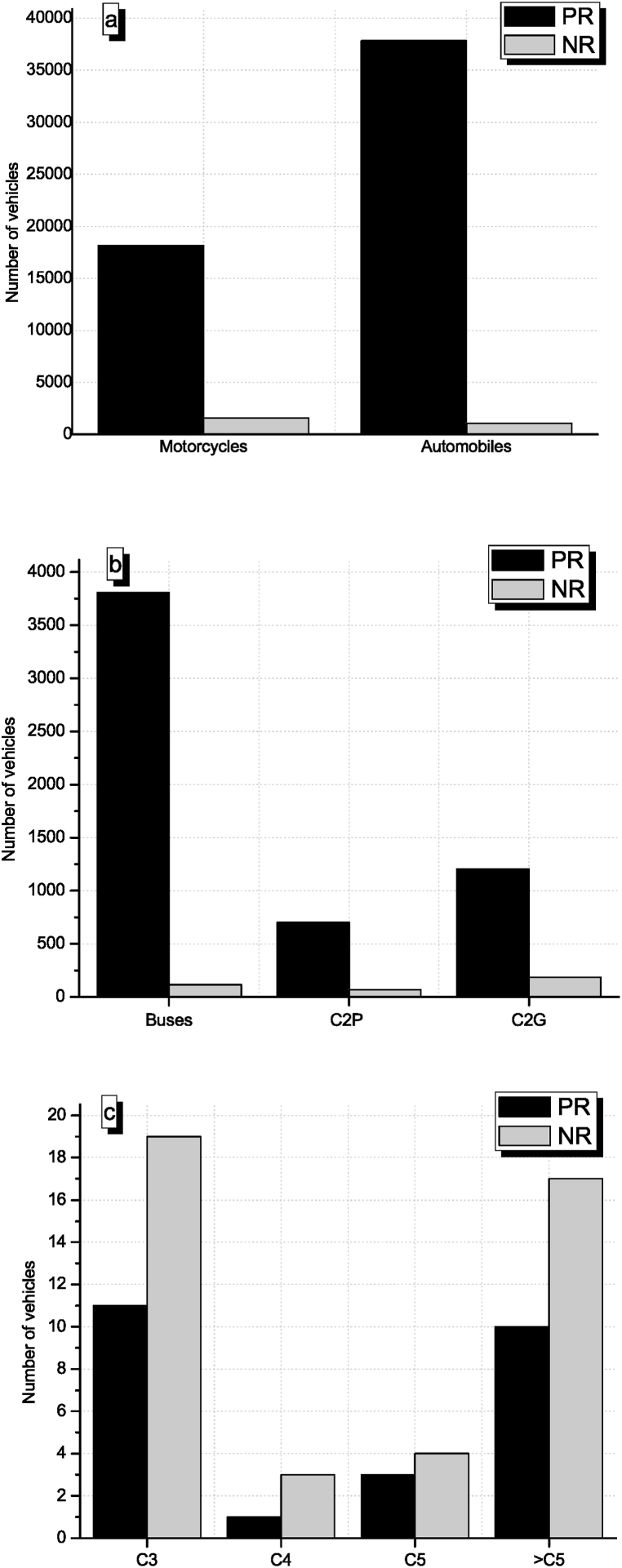
Number of motor vehicles by types in PR (black bars) and NR (grey bars) per week. a) motorcycles and automobiles, b) buses, small trucks (C2P-two axle), big trucks (C2G-two axle), c) Three-axle Truck (C3), Four-axle Truck (C4), Five-axle Truck (C5) and Six or more axle Truck (>C5).

Fig. 2 shows the weekly average of motorcycles, automobiles, buses, C2P, C2G, C3, C4, C5, and > C5 in PR and NR (Fig. 2 a-c). A significant difference between the number of light-duty and medium-duty motor vehicles was found between PR and NR (Fig. 2 a, b). The Average number of motorcycles, automobiles, buses, C2P, and C2G in PR was about 12, 36, 33, 10, and 6 times higher than in NR. On the contrary, heavy-duty trucks classified as C3, C4, C5, and > C5 had a higher presence, by a factor of 1.4, in NR compared to PR (Fig. 2c). However, in general terms, PR presented 20 times more motor vehicles than NR.

#### 2.2.2 Road features

The positive gradient and some variables related to track conditions of the road influence the PAH emission levels. Unlike PR, the transect chosen in NR presents a wide range of gradients (∼2.6–12.8) with an average speed rate between 13 and 16 m/s. Conversely, PR shows a high interval of speed (∼0–15 m/s) and a low variation in its gradient. Therefore, vehicle speed was not considered in NR, thus, road gradient was the most representative variable to be analyzed in this work. Likewise, variation in speed caused by the traffic control devices in PR has been considered.

Regarding road conditions, although both segments are paved, NR presents the worst circumstances in the entire road segment in the study, with the presence of holes, patches, fissures, and pavement settlement. However, this factor does not significantly influence the speed profile of motor vehicles.

### 2.3 Sample collection

Before the sampling process, multiple field campaigns were conducted for compiling the necessary information to define the experimental design adequately. This information included: tree data, lichen species, the incidence of pollutant sources; and thus, the selection of specific sampling sites. In addition, the sampling campaign was conducted after at least one week without precipitation records to not interfere with the bioaccumulation of PAHs in lichens.

For this work, the following inclusion criteria were defined to select the trees with lichens: diameter greater than 20 cm, trunk inclination less than 20° to the vertical, and absence of morphological damage to the bark, as recommended by Conti and Cecchetti (2001) [28]. Furthermore, the selected trees must have a minimum height of 1.5 m above the ground and the presence of lichen families of interest on their trunks [29]. About 40 trees in both study areas met these criteria.

The selected lichens belonged to the family Parmeliacea (*Hypotrachyna rhabdiformis (Kurok.*) *Hale*, *Parmotrema perlatum, Punctelia colombiana Sérus*), family Pannariaceae (*Parmeliella* sp.) and family Physciaceae (*Heterodermia obscurata (Nyl.) Trevis*) (see Fig. 1). These families have evidenced to be widely adapted to the environment of the study area and the intertropical zone. The samples comprised a balanced mixture of all species for each tree. The main reason for this decision is that there is a small amount of lichens of a single species per tree to be processed and obtain enough target analyte.

The lichens were identified considering some characteristics that specifically define each family, such as color, lobe shape, and growth form. Analyzed species were deposited in the herbarium of Universidad del Cauca (CAUP), a member of the Colombian Association of Herbariums (ACH). Lichen codes in the herbarium are between 53069 and 53073.

The sample size for each transect was determined by considering Cochran's formula (1), developed to estimate a representative sample when specific information about the population may be known, and a random sampling method is used to choose the individuals.

(1) $$n=\frac{z^{2}\times p\times \left(1-p\right)}{e^{2}}\text{.}$$

In this case, the parameters for equation (1) are a margin of error (e) of 5%, a confidence level of 95% (z:1.96) and the proportion of the population of trees with lichens (p) was 99%.

During the process, a number was assigned to each tree that met the inclusion requirements. Subsequently, they were chosen by random number selection.

A sampling and care protocol was defined to guarantee an adequate capture and preservation of the lichens. This protocol encompassed the usage of nitrile gloves and face masks to prevent the loss or contamination of lichens, in addition to a spatula, stainless-steel tweezers, surgical blade, and aluminum bags, previously sterilized in the laboratory to avoid cross contamination considering that PAHs are ubiquitous compounds.

Approximately 2 g of lichen were collected from each phorophyte and stored in aluminum bags. Its collection was conducted carefully by manually separating the lichen from the tree's bark using a spatula and stainless-steel tweezers. Thereafter, the samples were transported in Styrofoam cooler boxes to the laboratory, where they were preserved at −20 °C.

### 2.4 Analytical procedure

#### 2.4.1 Pre-treatment of lichen samples

During pre-treatment, impurities were manually removed from the lichens, e.g., soil, tree bark, insects, etc., to eliminate interferences in the analytical procedure. Afterwards, samples were subjected to a lyophilization process to dry and grind them.

Lyophilization procedure was applied to each sample for 18 h (lyophilizer brand Liobras, L101), following initial conditions of (T): −54 °C, voltage (V): 219 V, Pressure (P): 205 μmHg and final conditions of T: −58 °C, V: 219 V, P: 62 μmHg. This procedure is efficient in drying samples since it allows their dehydration at low temperatures reducing analyte loss. From that point, samples were ground in a ceramic mortar until they became a fine powder; and finally, they were stored in an amber container previously cleaned at muffle at 400 °C.

#### 2.4.2 Analytical method

The analytical method used for quantifying 16 PAHs combines the ultrasound-assisted extraction of the samples, evaporation, clean-up in a glass column, and analysis of the extract through gas chromatography coupled to mass spectrometry (GC-MS). A standard mix of the 16 PAHs (CRM48905, PAH Mix in dichloromethane: benzene 1:1, Supelco brand) and surrogate standard (*p*-terphenyl D14, in methylene chloride, Supelco brand) was used for building the calibration curve.

For the analytical process, around of 0.2 g of raw lichen of each species collected (Sp1-Sp6) was mixed and then subjected to a pre-treatment process until obtaining the powdered lichen. Thereupon, the extraction was carried out by triplicate using 0.4 g of the mixture of powdered lichen (dry weight) plus 40 μL of surrogate standard with a final concentration of 100 ng/g in the lichen. Additionally, 2 mL of hexane: dichloromethane (3: 2 v/v), both analytical grade (HPLC grade), were added. The sample, surrogate, and solvent were subjected to an ultrasound bath for 10 min (Unique brand) at room temperature. Following that, solid separation from the liquid medium was performed by a Macro IV centrifuge, model EV: 025, for 5 min at 3000 rpm. Finally, an aliquot of 2 mL of supernatant extract was taken for each triplicate, thus obtaining a combined extract of 6 mL, which was concentrated in a Christ brand rotary evaporator, model RVC 2–18 CDplus at 60 °C for 20 min until approximately 0.5 mL.

The clean-up was carried out by column adsorption chromatography, using a glass column as stationary phase packed with glass wool, 2 g of activated silica gel, 1 g of activated alumina at 130 °C, in a Fanem brand oven (model Orion 515), and 0.5 g of anhydrous sodium sulfate, according to EPA 3630B [30]. The mobile phase (15 mL) used was hexane: dichloromethane in a ratio of 3:2 (v/v). Moreover, a conditioning procedure was performed before cleaning, using the same solvent as the mobile phase, as shown by Ref. [31].

The collected samples were evaporated entirely in an evaporator at 60 °C for 30 min, then dissolved in 225 μL of dichloromethane and 25 μL of internal standard (100 ng/mL) (Semivolatile Internal Std Mix, CRM5M07296, in dichloromethane. Naphthalene-d8, acenaphthene-d10, phenanthrene-d10, chrysene-d12 and perylene-d12, Supelco brand). Sigma Aldrich supplied all reagents.

A gas phase chromatography technique coupled with mass spectrometry (GC-MS) was used to identify and quantify PAHs. Samples were analyzed using a Shimadzu model QP2010-TQ8040 triple quadrupole gas chromatograph with Shimadzu AOC5000 combi-PAL autosampler and GCMS-TQ8040 mass spectrometer. It was injected with 1 μL per sample, and thereafter, compounds were separated using a DB-5Ms capillary column (30 m × 0.25 mm x 0.25 μm) as the stationary phase. The helium supplied by White Martins (Paraná, Brazil) (99.999%) was used as carrier gas at a 1.20 mL/min flow rate.

Analytical standards and samples were injected in splitless mode, with an injector at 270 °C, without a pressure pulse and a sampling time of 4 min. The chromatographic column was initially kept at 50 °C for 5 min, followed by heating at 5 °C/min up to 230 °C, then gradient 2 °C/min up to 250 °C was used, and finally ramping at 5 °C/min up to 300 °C, held for 8 min. The transfer line and the ion source were operated at 280 °C and 230 °C, respectively. The mass spectrometer was configured in Selective Ion Monitoring (SIM) mode, which provides higher selectivity and sensitivity to the analysis.

#### 2.4.3 Quality control and quality assurance

Additional information on analytical methods, e.g., quality assurance and quality control of the analytical procedure, together with a limit of quantification (LOQ), the limit of detection (LOD), recoveries for each internal standard, and %RSD are provided elsewhere [32]. However, some details will be included here.

Recommendations by Refs. [[33], [34], [35]] were considered during sampling lichens, which reduced uncertainty and avoided problems such as those commented by Ref. [17]. Optimization of clean-up experimental conditions was conducted in two tests using a univariate analysis methodology to achieve a better stationary phase and proper solvent. Hexane-dichloromethane 3:2 (v/v) was the best solvent eluent, and ASG-AA performed better. Different weights of lichen powdered were evaluated; 0.4 g showed the best results.

The curves of the analytical method were done with 7 solutions in triplicate (PAH Mix- CRM48905, Supelco), showing R^2^ > 0.99. LOD and LOQ ranged from 1 to 5 μg/L and 3–15 μg/L, respectively. The RSD percentage was between 4.2% and 22.1% for most cases, showing good repeatability of the analytical method.

### 2.5 Carcinogenic equivalence sum (KE)

The present paper shows the Carcinogenic equivalence sum (KE) values for PAHs found in both places, PR and NR. It evaluates health risks due to PAH carcinogenic potential through Toxic Equivalent Factor (TEF) and concentration of individual compounds [36], calculated according to equation (2).

(2) $$KE=\sum_{i=1}^{16}TEF_{i}\times PAH_{i}\text{.}$$

Terms $TEF_{i},i=1,\ldots ,16$ take values between 0 and 1, indicating PAH toxicity level with respect to B(*a*)P, which has the highest value. Different values for TEF have been proposed, being the values presented by USEPA (1993), Cecinato (1997), and Nisbet and LaGoy (1992) the most common ones [[37], [38], [39], [40]]. A detailed description of KE and TEF can be found in Ref. [36].

### 2.6 Statistical analysis

Normality and correlation tests were conducted to analyze the PAH data trend statistically. The results for normality showed a significance of less than 0.05 in the Kolmogórov-Smirnov test (MATLAB software), which indicates a non-normal distribution. When data does not follow a normal distribution, non-parametric methods should be used. Hence Spearman's correlation test was chosen. Spearman allowed evidence if there was a correlation between road gradient and PAHs associated with lichens. Spearman's rho (R) measured the strength of the association between the two variables [41].

## 3 Results

### 3.1 Concentration of PAHs in NR and gradient

The NR was divided into six intervals of distances based on marked changes in road gradients. The total PAH concentration (∑_16_PAHs) in each road interval was calculated as the average of samples belonging to that interval (Table 2).

**Table 2:** The intervals of road and samples clustered in NR.

Interval of road	Averaged samples^1^
1	S1, S2
2	S3 to S8
3	S9 to S11
4	S12, S13
5	S14
6	S15

^1^S1–S15: Samples of lichen in NR. S1 corresponds to sample number one, and so on.

PAH level in each sample (S1–S15) was included in Table S1 of supplementary materials.

**Fig. 3:**
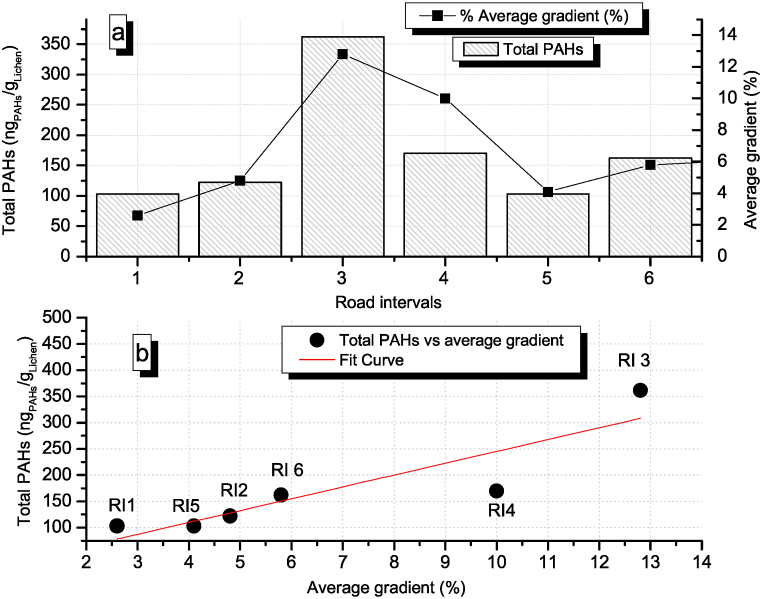
Total PAHs in relation to road gradient in NR. a) x axis: Intervals of distances in NR, bars: total PAH concentration in each segment (ng_PAHs_/g_Lichen_), black squares: road average gradient. b) fit curve for the regression between total PAH concentration vs. average road gradient (Spearman R=0.98,p<0.005,n=6) with the fitting formula y=22.586⋅x+19.383. RI1 to RI6: road intervals.

Fig. 3a shows the intervals with the average of total PAH concentration in each road gradient. Fig. 3b presents the fitting curve for the regression between total PAH concentration as a function of the road average gradient (Spearman R=0.98,p<0.005,n=6). These results indicate a linear correlation between the variables.

PAH concentrations are tracking the behavior of the road gradient, with a maximum value of 362 ng_PAH_/g_Lichen_ in interval 3 with an average gradient of 12.8%. Outcomes evidence an increase of about 24 ng_PAH_/g_Lichen_ per every 1% increase in road gradient.

### 3.2 Concentration of PAHs in PR and vehicle speed

**Fig. 4:**
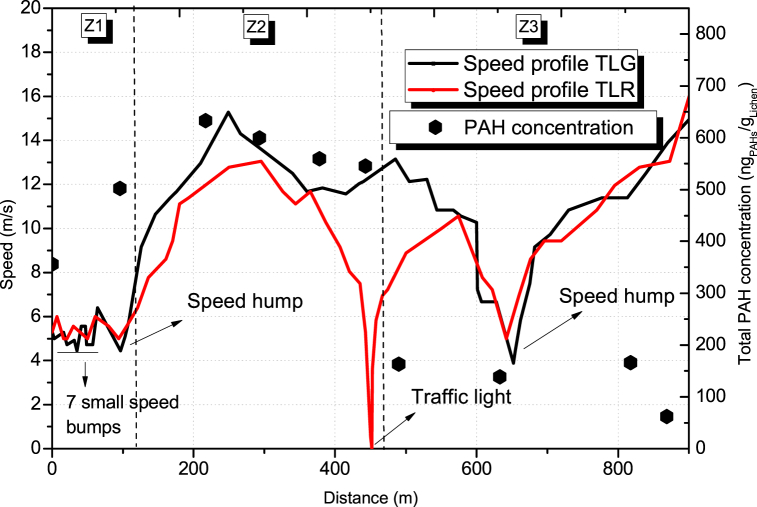
Relationship between vehicle speed and total PAH concentration for different distances in PR. The distance at zero meters is related to the location of the first sample. Due to their proximity, samples in distances 0 m, 96 m, and 490 m were clustered from 3, 3 and 2 samples. The PAH level of each sampling site was included in the supplementary material (Table S2).

The gradient in PR (<3% on average) was not considered as an influencing variable on PAH emission since the road is flat. Conversely, the vehicle speed varied significantly due to the presence of the speed control devices, so this variable was considered for the study. As shown in Fig. 4, three different segments (Z1, Z2, and Z3) can be identified on this road based on patterns for vehicle speed with traffic lights in green (TLG) or red (TLR) and total PAH concentration. This fact was due to the location of small speed bumps, speed humps, and traffic lights (see Table 1).

In Fig. 4, PAH concentration (black hexagon) seems to be a function of vehicle speed in PR. Two trends of concentration levels can be observed. On the one hand, before the traffic light, a high concentration (570 ± 79 ng_PAHs_/g_Lichen_ in average) of PAHs associated with lichens was shown with the distance, except for the first sample with a minor concentration (356 ng_PAHs_/g_Lichen_). On the other hand, after the traffic light, the concentration of PAHs decreases to around 139 ± 16 ng_PAHs_/g_Lichen_, with an endpoint of 62 ng_PAHs_/g_Lichen_.

### 3.3 Individual concentration of PAHs in NR and PR

**Fig. 5:**
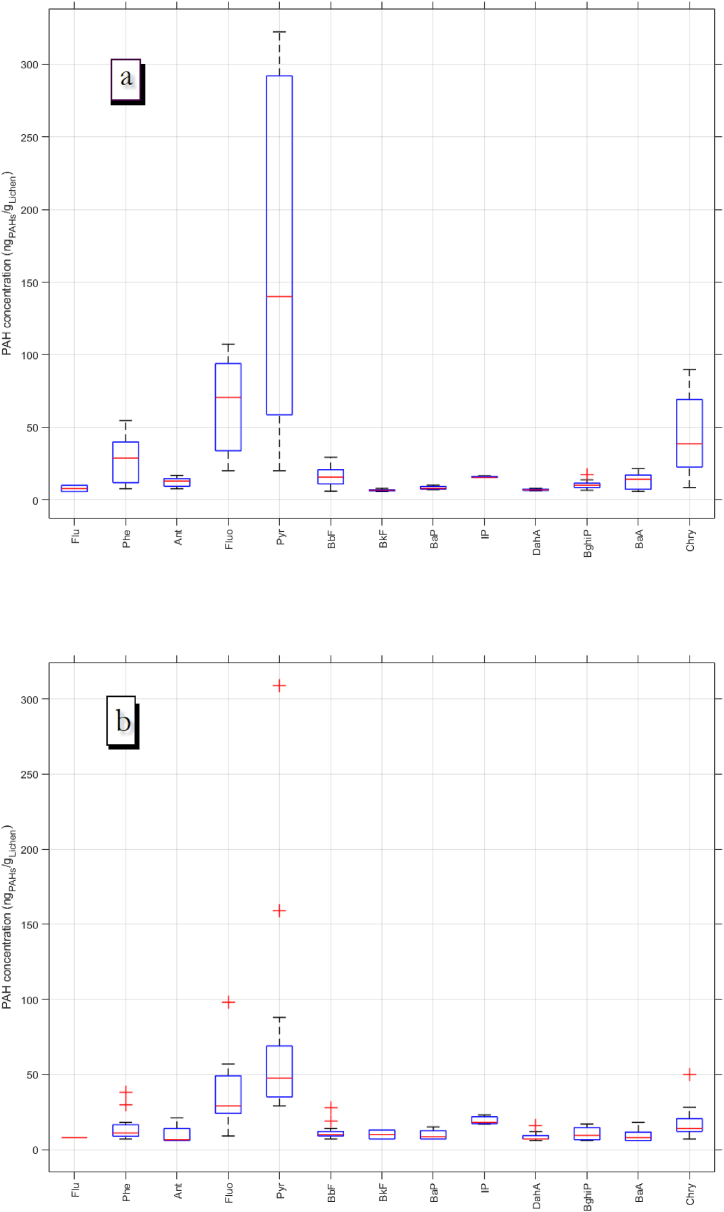
Box plots of individual concentrations of PAH found in lichen for the different samples (n = 15). a) PR and b) NR.

Fig. 5 shows the box plots for PAH concentration associated with lichens in trees placed in PR (n = 15) (Fig. 5a) and NR (n = 15) (Fig. 5b). PAHs as Nap, Ace, and Acy were found to be below the LOD of the method, which corresponds to compounds with 2 and 3 aromatic rings with higher volatility and lower persistence.

Data with high dispersion was found in PR for aromatics with higher concentration, showing that the speed control measures in PR and traffic volume cause a significant variation in the emission of certain aromatics rather than changes in gradient, as it is the case of NR.

### 3.4 Carcinogenic equivalent sum (KE)

**Fig. 6:**
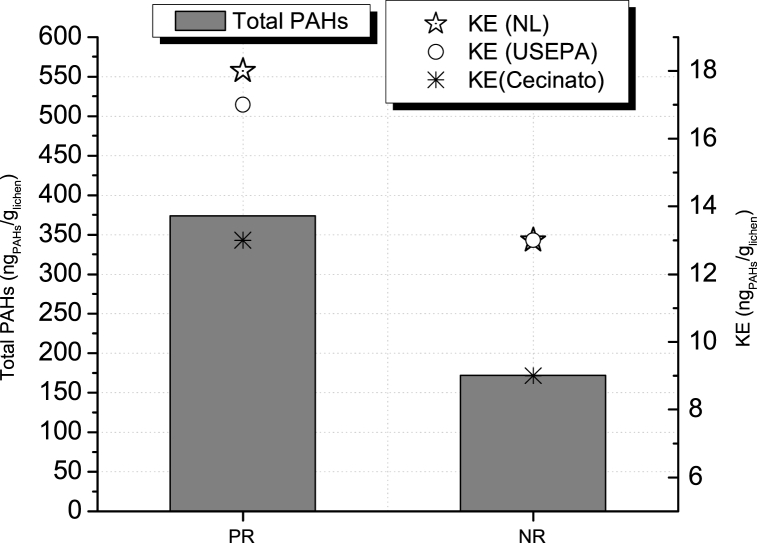
Average of PAHs in study areas and average KE (ng_PAHs_/g_Lichen_). KE were determined by using different TEF values, starts: TEF from (Nisbet and Lagoy 1992), circles: TEF from (USEPA, 1993), asterisk: TEF from (Cecinato, 1997). Bars: Total PAHs.

The carcinogenic equivalence sum was used to evaluate the capacity of lichens to provide an insight of the level of carcinogenic potential of PAHs bio-accumulated as an indicator of their presence in the atmosphere. Fig. 6 shows the average of total PAHs (grey bars) for all points and KE (stars, circle, and asterisk according to TEF evaluated) in both study areas.

Total PAH concentration found in PR (374 ng_PAHs/_g_Lichen_) was 117% higher than NR (172 ng_PAHs_/g_Lichen_).

## 4 Discussion

### 4.1 Comparison of PAH concentrations in NR and PR relative to road characteristics and traffic patterns

The difference in total PAH concentration between NR and PR is attributed to vehicle capacity of the road or higher number of vehicles passing in the transect per day, mainly motorcycles and automobiles. A study on vehicle emissions of PAH found that light-duty vehicles and motorcycles are significant contributors to PAHs emissions mainly due to their large population [42]. Alexandrino et al., 2022 [6] evaluated the PAHs associated with *Araucaria heterophylla needles* at different urban areas. This work showed that the road capacity has a greater influence on the total concentration of PAHs.

Total concentration of PAHs in NR is comparable to vehicle traffic emission levels found in a national highway with sloped characteristics in Tibetan Plateau, which ranged between 3.29 and 119 ng/g DW [43]. However, the concentrations observed in this study are lower than those reported by Ref. [6] in an urban area of Quito (520–9230 ng_PAHs_/g_Araucaria_ DW). It is worth noting that Quito has similar geographical conditions to Popayán, but with a higher population and consequently, more intense vehicular activity, which contribute to higher PAH level in urban area.

A Spearman correlation analysis was implemented to NR data in order to identify strength degree of relationship between PAHs concentration and road average gradient. The analysis revealed a strong and statistically significant correlation (p<0.005) with Spearman's rho of R=0.98. These results suggest that road average gradient has a strong influence on fuel consumption; and consequently, on PAH emissions in the studied tracks. Fuel consumption; and therefore, pollutant emissions were significantly higher on steep roads than on flat roads as was previously reported in Ref. [44]. Boriboonsomsin and Barth (2009) [45] found that fuel consumption could decrease about 15%–20% in flat routes compared to steep roads, although this depends on factors such as vehicle weight and vehicle load.

The presence of traffic control devices (traffic lights, speed bumps, etc.) in PR caused a specific vehicle speed profile, with a significant drop observed in two segments, mainly due to speed reducers (SSB, BSH) (Z1 and Z3) and an intermittent stop caused by traffic light in Z2. As observed in the present study, the literature shows a speed reduction in the range of 18–75% in roads using BSH [10,11].

Polycyclic aromatic hydrocarbon concentrations in Z1 increase possibly due to the stopping of the vehicles and subsequent fuel consumption by acceleration. This behaviour was noted after a big speed hump. The increase of particulate matter due to the presence of speed reducers had been ranged from 45% to 75% in a previous work [46]. A similar value was observed in this work, as PAHs increased by 41% in Z1. Although the present work refers to PAH concentration, there was a strong positive correlation between PM with diameter less than 2.5 μm and aromatic compounds; thus, PAH increases effectively when PM does [47]. Unfortunately, the results here cannot be analyzed in comparison with other studies focused on the increase of PAH emissions with speed reducers provided that no literature has been found on this subject.

The concentration of PAHs in the second flat segment of 350 m (Z2) shows an increase probably due to rate of acceleration of the vehicles to catch the green light of traffic light, or/and posterior speed reduction for the red light when fail to do so. In this last case, they encounter a stop of about 37 s with vehicle engine running and subsequent start-up. According to Ref. [9], usually vehicular fuel consumption at signalized intersections was considerably higher than on regular segments. This fact could directly impact the background air quality, following the dynamics of dispersion of plume emissions given the location of motor exhaust pipe. This phenomenon in PR could be enhanced by wind speed like described by Ref. [8].

The concentrations of PAHs decrease in Z3 by about 77% in comparison to the average concentration found in Z2. This behaviour was observed just after the traffic light, even when a speed hump was placed nearby. The most noticeable difference between segments Z3 and Z1-Z2 is the landscape, characterized by presence of sparse tall trees on one side and dense shrubby vegetation on wide-open road on the other side. Some studies as [48] suggest that open road conditions, wide road and dense/tall vegetation lead to pollutant reductions with a positive impact greater than 50%. This reduction was principally due to the dense and tall vegetation (with a 10 m thick belt) at sites close to the pollutant source and at the maximum plume concentration. This factor could explain the reduction of PAHs in Z3 segment; nevertheless, additional studies considering other influencing (e.g., wind speed and temperature) variables should be conducted so as to achieve a comprehensive understanding of the trends here observed in different segments, especially Z3.

### 4.2 Individual concentration of PAHs in NR and PR

According to analysis of individual PAHs, thirteen out of sixteen PAHs were found in both study areas with a predominance of Fluo, Pyr, Chry, and Phe, as seen in Fig. 5 (a, b), whose prevalence has also been reported in previous studies on emissions related to vehicle traffic [49,50]. This behaviour was common to both roads, except for IP, which was found to have a majority in NR, an aromatic related to diesel vehicles exhaust.

Individual or isomer ratios of emitted PAHs have been used frequently in papers as tracers to identify their sources (Table 3). For instance, compounds with three aromatic rings, are essentially associated to heavy-duty diesel engines [51]. In contrast, light-duty vehicles are the major source of four- and five-ring compounds, correlated mainly with gasoline [52]. The latter is predominant in PR with 82% of the total PAHs, coinciding with the higher traffic of light-duty vehicles compared to NR (74%). This fact is confirmed by relation B[*a*]A/(B[*a*]A/Chry), whose ratio was 0.36 in PR and 0.19 in NR, indicating vehicular emissions with predominance of gasoline combustion in PR and from petrogenic origin in NR [53].

**Table 3:** Evaluation of PAH sources based in diagnostic ratios.

Dignostic ratio of PAHs	Present work in NR	Present work in PR	Diagnostic value	Reference
B[*a*]A/(B[*a*]A/Chry	0.19	0.36	<0.2 petrogenic 0.2–0.35 coal combustion >0.35 vehicular emission 0.33–0.38 gasoline combustion	[54] and references therein
Fluo/(Fluo + Pyr)	0.33	0.28	<0.5 fossil fuel comb. >0.5 grass, wood, coal	[54,55] and references therein
Ant/Ant + Phe	0.41	0.31	<0.1 petrogenic >0.1 pyrogenic or dominance of combustion.	[54] and references therein
BbF/BkF	7.7	2.4	>0.5 Diesel combustion	[54] and references therein
BaA/Chry	0.4	0.3	0.3–1.2 gasoline exhausts 0.2–0.4 diesel exhausts 0.7–0.9 wood combustion 1.0–1.2 coal/coke	[47] and references therein
BaP/Chry	0.19	0.19	0.4 gasoline exhausts ≈1.0 diesel exhausts	[47] and references therein

Ratio of Ant/Ant + Phe >0.1 points to the predominance of combustion in PR and NR by means of pyrogenic sources. Similar results were found by Ref. [56] in urban, suburban and rural sites monitoring total suspended particles (TSP). Other ratios such as BbF/BkF, BaA/Chry and BaP/Chry [47] also indicate vehicular emission sources from both diesel and gasoline engines in both study sites.

The ratio PAHcomb/PAHtotal ≈ 0.70 found in both roads also seems to evidence diesel/gasoline engines as the source. In this case, PAHcomb refers to compounds closely related to combustion sources; they are PAHcomb: Fluo + Pyr + BaA + Chry + BbF + BkF + BaP + DahA + BghiP. Similar ratio was found by Ref. [57] in samples of pine needles from Mexico City, what may be the results of intensive combustion activity as mentioned by authors.

In general terms, through the information obtained from the evaluation of the individual aromatic ratios, the present study provides evidence on the usefulness of lichens in the determination of aromatic sources. This is due to the fact that ratios of PAH concentration measured in lichens pointed to gasoline and diesel combustion processes, the main pollution source in this work.

### 4.3 Carcinogenic equivalence sum (KE)

Carcinogenic equivalence sum was found to be around 20%–30% higher in PR than in NR for all TEF presented by USEPA (1993), Cecinato (1997) and Nisbet and LaGoy (1992) [[37], [38], [39], [40]]. However, even though vehicle fleet and PAH concentration in PR were 20.4 and 2.2 times higher, respectively than NR, KE values do not differ in the same proportion (1.3 times higher PR than NR). This can be explained by the fact that PAH compounds considered as having the highest carcinogenic potential; and thus, TEF values (IP, BaP, DahA and BkF) presented higher concentrations in NR, this fact was addressed by Ref. [36]. In other words, the results suggest that the presence of a large number of vehicles with diesel engine on the road may increase the carcinogenic potential of PAHs, given the prevalence of emissions including PAH with higher toxicity factors.

## 5 Conclusions

PAH concentrations associated to lichens in a city road (PR) and national road (NR) with different road features were analyzed. The purpose was to determine the capabilities of lichen to capture levels of PAHs in the atmosphere associated to emissions related to speed modifying characteristics of roads, emission sources, and carcinogenic potential.

Intensive vehicular traffic fuelled by fossil fuels could be an indicator of the presence of potential carcinogens, especially in low-middle income countries. Nevertheless, a road with less traffic does not necessarily guarantee clean air, since types of vehicles, geometric design features, among others could favor the carcinogenic effect of emissions. To determine this, as observed in the present work, a reliable and cost-effective method may be the analysis of PAHs associated to lichens in phorophytes near emission sources.

The strong correlation between PAHs (measured by GC-MS chromatography from lichens collected from roads) and road gradient, as well as the apparent relationship between traffic control devices suggest that photobionts are reliable for monitoring traffic-related PAHs and sources. The profile of PAH concentration associated to lichens in PR road followed the expected trend marked by the variation of vehicle emissions, caused in turns by the presence of speed reducer devices. In addition, gradient and PAH concentration were significantly correlated in NR. Additionally, ratios of PAHs pointed to vehicle traffic fuelled by gasoline and diesel as main source, what is completely right for this work.

The information provided here constitutes some initial evidence on potential and cost-effective tool for the assessment of PAH concentration, which could facilitate the monitoring of target areas, the implementation of control strategies in relation to impact on the environment and human health. However, authors encourage to conduct further studies to complement this work and to evaluate additional features related to traffic emissions since very little literature information was found to critically achieve a deeper analysis to the outcomes presented here.

## Funding

This research did not receive any specific grant from funding agencies in the public, commercial, or not-for-profit sectors.

## Author contribution statement

Diana Marcela Uribe; Lina María Ortega: Conceived and designed the experiments; Performed the experiments; Contributed reagents, materials, analysis tools or data.

Marco Tadeu Grassi; Rafael Garrett Dolatto: Conceived and designed the experiments; Analyzed and interpreted the data; Contributed reagents, materials, analysis tools or data.

Nazly Sanchez: Conceived and designed the experiments; Performed the experiments; Analyzed and interpreted the data; Contributed reagents, materials, analysis tools or data; Wrote the paper.

## Data availability statement

Data included in article/supplementary material/referenced in article.

## Declaration of competing interest

The authors declare that they have no known competing financial interests or personal relationships that could have appeared to influence the work reported in this paper.
