# Supplementary material for: Lichens as bio-monitors of polycyclic aromatic hydrocarbons: Measuring the impact of features and traffic patterns
Source: Heliyon. 2023 Sep 13;9(9):e20087. doi: 10.1016/j.heliyon.2023.e20087 (PMC10559864; doi:10.1016/j.heliyon.2023.e20087)
Supplement: Multimedia component 1 [file mmc1.docx]

Supplementary material

Table S1. Total PAH concentration in samples from NR

| **Samples** | **Total PAH concentration (ng_PAHs/_g_Lichen_)** |
| --- | --- |
| S1 | 109 |
| S2 | 97 |
| S3 | 16 |
| S4 | 99 |
| S5 | 133 |
| S6 | 117 |
| S7 | 136 |
| S8 | 232 |
| S9 | 316 |
| S10 | 614 |
| S11 | 155 |
| S12 | 170 |
| S13 | 103 |
| S14 | 162 |
| S15 | 116 |

Table S2. Total PAH concentration in samples from PR

| **Samples** | **Total PAH concentration (ngPAHs/gLichen)** |
| --- | --- |
| S1 | 597 |
| S2 | 294 |
| S3 | 176 |
| S4 | 595 |
| S5 | 542 |
| S6 | 370 |
| S7 | 633 |
| S8 | 600 |
| S9 | 559 |
| S10 | 545 |
| S11 | 180 |
| S12 | 146 |
| S13 | 139 |
| S14 | 166 |
| S15 | 62 |
